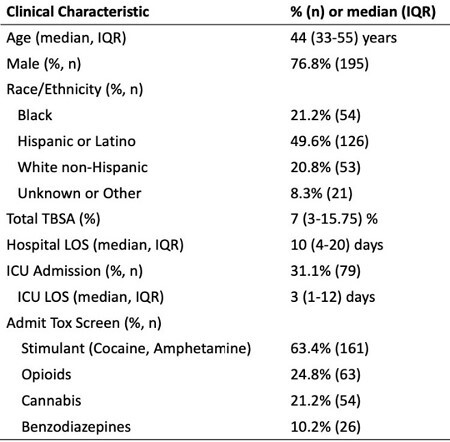# 742 Injury Pattern Analysis to Optimize Burn Injury Prevention in the Unhoused Community

**DOI:** 10.1093/jbcr/irae036.285

**Published:** 2024-04-17

**Authors:** Erin E Ross, Noah Speiser, Sean J Donohue, Justin Gillenwater, Haig A Yenikomshian

**Affiliations:** Keck School of Medicine of USC, Los Angeles, CA; Keck School of Medicine, University of Southern California, Rolling Hills, CA; University of Southern California, Studio City, CA; Keck Medicine of USC, Los Angeles, CA; University of Southern California, Los Angeles, CA; Keck School of Medicine of USC, Los Angeles, CA; Keck School of Medicine, University of Southern California, Rolling Hills, CA; University of Southern California, Studio City, CA; Keck Medicine of USC, Los Angeles, CA; University of Southern California, Los Angeles, CA; Keck School of Medicine of USC, Los Angeles, CA; Keck School of Medicine, University of Southern California, Rolling Hills, CA; University of Southern California, Studio City, CA; Keck Medicine of USC, Los Angeles, CA; University of Southern California, Los Angeles, CA; Keck School of Medicine of USC, Los Angeles, CA; Keck School of Medicine, University of Southern California, Rolling Hills, CA; University of Southern California, Studio City, CA; Keck Medicine of USC, Los Angeles, CA; University of Southern California, Los Angeles, CA; Keck School of Medicine of USC, Los Angeles, CA; Keck School of Medicine, University of Southern California, Rolling Hills, CA; University of Southern California, Studio City, CA; Keck Medicine of USC, Los Angeles, CA; University of Southern California, Los Angeles, CA

## Abstract

**Introduction:**

Unhoused patients represent a unique and vulnerable population of burn survivors. Burn injuries in unhoused patients are more likely to be related to assault, drug use, and mental health issues compared to the general population. Here, we explore the more specific circumstances and activities contributing to burn admissions among unhoused patients to better inform injury prevention efforts.

**Methods:**

After IRB approval, the burn registry at an urban regional burn center was queried for burn admissions in unhoused adults from 2019-2022. Registry data pulled included demographics, urine toxicology, mechanism of injury, and injury subjective history. Subjective injury history was reviewed to determine more specific injury circumstances and activities during which accidental burns occurred. Demographic and mechanistic trends in burn admissions were explored via descriptive statistics.

**Results:**

There were 254 admissions to the burn unit for unhoused patients. Patients were mostly male (76.8%), Hispanic (49.6%), and middle aged (IQR 33-55 years). 58.1% of patients were positive for stimulants on admission. The most common burn etiologies were open flame (27.6%), burning clothing or bedding (17.7%), and accelerants (14.2%). Most burns were self-reported accidental (69.7%), 9.8% assault, and 7.1% self-inflicted (missing = 34). Among accidental injuries, 11.3% were related to cooking, 10.1% involved cooking or using methamphetamine, 9.6% involved smoking, 7.3% were due to bonfires, and 5.6% were caused by candles.

**Conclusions:**

Most burn injuries among unhoused patients were accidental. Although methamphetamine use was directly related to only a small proportion of accidental injuries, more than half of unhoused patients were positive for stimulants upon admission. Most burn injuries among unhoused patients were preventable, with many accidental injuries related to cooking, smoking, and open flames for warmth or light.

**Applicability of Research to Practice:**

Analysis of admission patterns and subjective history can reveal potential interventions for preventable causes of injury. Safe consumption sites, as well as outreach efforts to educate unhoused patients about situational awareness, accelerants, safe smoking practices, and safe cooking practices may be effective tools in reducing burn admissions in this vulnerable population.